# Fluid balance and cardiac function in septic shock as predictors of hospital mortality

**DOI:** 10.1186/cc13072

**Published:** 2013-10-20

**Authors:** Scott T Micek, Colleen McEvoy, Matthew McKenzie, Nicholas Hampton, Joshua A Doherty, Marin H Kollef

**Affiliations:** 1 St. Louis College of Pharmacy, 4588 Parkview Place, St. Louis, MO 63110, USA; 2 Division of Pulmonary and Critical Care Medicine, Washington University School of Medicine, 660 South Euclid Avenue, Campus Box 8052, St. Louis, MO 63110, USA; 3 BJC Center for Clinical Excellence, 4901 Forest Park Avenue, St. Louis, MO 63108, USA; 4 Medical Informatics, BJC Learning Institute, 8300 Eager Road, St. Louis, MO 63144, USA

## Abstract

**Introduction:**

Septic shock is a major cause of morbidity and mortality throughout the world. Unfortunately, the optimal fluid management of septic shock is unknown and currently is empirical.

**Methods:**

A retrospective analysis was performed at Barnes-Jewish Hospital (St. Louis, Missouri). Consecutive patients (n = 325) hospitalized with septic shock who had echocardiographic examinations performed within 24 hours of shock onset were enrolled.

**Results:**

A total of 163 (50.2%) patients with septic shock died during hospitalization. Non-survivors had a significantly larger positive net fluid balance within the 24 hour window of septic shock onset (median (IQR): 4,374 ml (1,637 ml, 7,260 ml) vs. 2,959 ml (1,639.5 ml, 4,769.5 ml), *P* = 0.004). The greatest quartile of positive net fluid balance at 24 hours and eight days post-shock onset respectively were found to predict hospital mortality, and the greatest quartile of positive net fluid balance at eight days post-shock onset was an independent predictor of hospital mortality (adjusted odds ratio (AOR), 1.66; 95% CI, 1.39 to 1.98; *P =* 0.004). Survivors were significantly more likely to have mild left ventricular dysfunction as evaluated by bedside echocardiography and non-survivors had slightly elevated left ventricular ejection fraction, which was also found to be an independent predictor of outcome.

**Conclusions:**

Our data confirms the importance of fluid balance and cardiac function as outcome predictors in patients with septic shock. A clinical trial to determine the optimal administration of intravenous fluids to patients with septic shock is needed.

## Introduction

Septic shock is a common disorder faced by clinicians working in the ICU setting. Intravenous fluids, along with appropriate antibiotic therapy, source control, vasopressors, inotropes and ventilator support are key elements of the management of septic shock [1]. The administration of intravenous fluids is largely empiric, although goal-directed approaches have been evaluated in an attempt to optimize fluid resuscitation of septic shock [2-4]. It is now recognized that excessive fluid administration in septic shock may contribute to acute lung injury (ALI), abdominal compartment syndrome, coagulopathy and cerebral edema [5-8]. We previously demonstrated that both early and late fluid management of septic shock complicated by ALI may influence patient outcomes [9]. Other investigators have recently demonstrated that positive net fluid balance is associated with increased risk of mortality in septic shock [10,11]. Moreover, the presence of cardiac dysfunction resulting from septic shock may also be an important predictor of outcome, although not all studies are in agreement on this point [12-14]. Therefore, we set out to perform a study with two main goals. The first study goal was to determine the relationship between net fluid balance and hospital mortality in a well described cohort of patients with septic shock. The second study goal was to assess whether the identification of newly recognized cardiac dysfunction influenced cumulative fluid balance or hospital mortality in patients with septic shock.

## Materials and methods

### Study location and patients

The study was conducted at Barnes-Jewish Hospital/Washington University Medical Center (1,300 beds) in St. Louis, MO and approved by the Institutional Review Board of Washington University. Patients with septic shock having a transthoracic echocardiographic examination performed within 24 hours of the onset of septic shock between 1 January 2009 and 31 December 2011 were eligible for this investigation. Patients were excluded if they had known pre-existing non-sepsis related cardiovascular compromise as defined by acute myocardial infarction, cardiogenic shock or a history of congestive heart failure with a left ventricle ejection fraction (LVEF) less than 40%; had a requirement for extracorporeal membrane oxygenation or a ventricular assist device; or developed septic shock at an outside hospital requiring vasopressor and fluid management prior to transfer.

### Study design

A retrospective cohort study was performed with the primary outcome being hospital mortality. Secondary outcomes included ICU and hospital length of stay, total quantity of intravenous and enteral fluids administered and the prescription of appropriate initial antimicrobial treatment. For the purposes of determining compliance with early, goal-directed treatment guidelines and the timing of antibiotic administration, the time of septic shock onset was defined as the time that a vasopressor agent was first administered. All pertinent data were then collected relative to this time.

### Data collection

Patients with septic shock were identified electronically by ICD9 codes for acute organ dysfunction and acute infection and by an active order for a vasopressor through the pharmacy database at Barnes-Jewish Hospital [15]. Data were collected retrospectively from automated patient medical records and pharmacy databases at Barnes-Jewish Hospital (MM, STM). Pertinent demographic, laboratory and clinical data were gathered including: age, gender, race, patient location at the time of septic shock onset, severity of illness based on the Acute Physiology and Chronic Health Evaluation (APACHE) II score [16], co-morbidities, Charlson co-morbidity score, site of the infection and positive cultures with sensitivities. Patient-specific factors starting at the time of septic shock onset were also collected including vital signs, central venous pressure (CVP), central venous hemoglobin oxygen saturation (S_CV_O_2_) and laboratory data. Information regarding the management of septic shock was recorded including adequate initial fluid resuscitation (AIFR), appropriate antimicrobial administration, corticosteroid administration and daily fluid balance.

### Definitions

Septic shock was defined as noted above by an ICD9 code for acute organ dysfunction (for example, acute renal failure, respiratory failure) in the presence of an acute infection and by an active order for a vasopressor that was administered for greater than 12 hours [15]. Onset of septic shock was defined as the time of vasopressor initiation. AIFR was defined as the administration of an initial fluid bolus ≥20 mL/kg and achievement of a CVP ≥8 mm Hg within eight hours after the onset of therapy with vasopressors. This was dictated by the hospital’s sepsis protocol and order set applied to all patients, as was the early use of vasopressors in patients with septic shock [17,18]. Appropriate empiric antimicrobial therapy was defined as antimicrobials given within 24 hours of the onset of septic shock that were active against the pathogen associated with infection based on susceptibility testing [19].

### Echocardiographic evaluation and definitions of myocardial dysfunction

Transthoracic echocardiography was performed in the ICU by certified echosonographers with a commercial instrument (Vivid I, GE Medical Systems, Milwaukee, WI, USA). All echocardiograms were interpreted by board-certified cardiologists from Washington University School of Medicine Cardiovascular Division. A comprehensive M-mode, two-dimensional and Doppler echocardiographic study was performed from the parasternal long- and short-axis views; apical four-chamber, two-chamber and long-axis views; and subcostal views.

LV end-diastolic volume, LV end-systolic volume, and LVEF using the modified Simpson method were assessed as recommended by the American Society of Echocardiography [20]. Measurements were taken during three cardiac cycles and then averaged. Systolic dysfunction was defined as mild (LVEF, 45% to 54%), moderate (LVEF, 30% to 44%), and severe (LVEF, <30%). Whenever suboptimal endomyocardial border definition was encountered for volumetric assessment, M-mode imaging and expert visual estimation by the interpreting cardiologist determined the final LVEF. Diastolic function evaluation was performed in accordance with the American Society of Echocardiography guidelines and graded as absent or present with or without evidence of increased filling pressures [21]. A multimodal approach was used to evaluate for right ventricle (RV) dysfunction, which was graded as mild, moderate or severe. Lateral tricuspid annulus peak systolic velocity measured by tissue Doppler imaging (TDI) was used in association with the relative RV-to-LV size, motion of the RV wall, and expert evaluation by the interpreting cardiologist [22]. RV peak systolic velocity less than 15 cm/s was considered diminished lateral RV systolic motion consistent with RV dysfunction.

### Statistical analysis

The primary data analysis compared hospital survivors to hospital non-survivors. Continuous data were reported as the mean ± SD for parametric data and the median with interquartile ranges for non-parametric data. The Student’s *t*-test was used when comparing parametric data and the Mann–Whitney *U* test was employed to analyze non-parametric data. Categorical data were expressed as frequency distributions, and the Chi-squared test was used to determine if differences existed between groups. After univariate analysis, stepwise multivariable logistic regression was undertaken to determine independent risk factors for hospital mortality. Risk factors significant at the 0.10 level in the univariate analysis were included in the models, with the exception of cardiac function parameters which were included regardless of the univariate *P-*values. All tests were two-tailed, and a *P-*value <0.05 was determined to represent statistical significance. Cox regression analysis stratified according to fluid balance quartiles was used to adjust for the confounding effects of age, severity of illness and use of vasopressin. Analyses were performed using SPSS, version 11.0 for Windows (SPSS, Inc., Chicago, IL, USA).

In addition, the influence of fluid balance on hospital mortality was further estimated using propensity scores. In our study, propensity scores were estimated by fitting a logistic regression. The covariates included in the propensity score model were those with a potential impact on outcome: age, body mass index, Charlson comorbidity score, gender, APACHE II, mechanical ventilation, chronic obstructive pulmonary disease, coronary artery disease, chronic renal disease, cirrhosis, underlying malignancy, diabetes, LV dysfunction, RV dysfunction, diastolic dysfunction and LVEF. Propensity score quintiles were derived, and boxplots of the estimated propensity scores for the highest and lowest quartiles of fluid balance within each quintile of the propensity scores were plotted to assess the validity of the analysis. Finally, we fitted a logistic model for hospital mortality including as covariates the propensity score and fluid balance.

## Results

### Patients

A total of 325 consecutive patients were included in the study, of whom 162 (49.8%) survived and 163 (50.2%) died during hospitalization. Hospital non-survivors were statistically older, had greater severity of illness measured by APACHE II scores, and were more likely to have required mechanical ventilation (Table 1).

**Table 1:** Baseline characteristics

**Variable**	**Survivors**	**Nonsurvivors**	***P-*** **value**
	**N = 162**	**N = 163**	
Age, yrs:	58.5 ± 14.6	63.0 ± 14.0	0.005
Male, n(%):	75 (46.3)	73 (44.8)	0.784
Race, n(%):			
Caucasian	107 (66.0)	121 (74.2)	0.107
African-American	50 (30.9)	41 (25.2)	0.252
Other	5 (3.1)	1 (0.6)	0.121
Body mass index ≥ 40, n(%):	30.6 ± 10.9	29.7 ± 9.7	0.552
Charlson comorbidity score:	3.4 ± 3.3	4.4 ± 3.2	0.111
Coexisting conditions, n(%):			
Coronary artery disease	18 (11.2)	9 (5.5)	0.072
Chronic obstructive lung disease	42 (26.1)	39 (23.9)	0.701
Cirrhosis	29 (17.9)	37 (22.7)	0.335
Chronic kidney disease	29 (17.9)	22 (13.5)	0.290
Diabetes	43 (26.5)	39 (23.9)	0.611
Active malignancy	21 (13.0)	28 (17.2)	0.353
APACHE II:	21.7 ± 6.3	25.1 ± 6.7	<0.001
Mechanical ventilation, n(%):	114 (70.4)	140 (85.9)	0.001
Bloodstream infection, n(%):	29 (17.9)	34 (20.9)	0.575

Values are expressed as mean ± SD. APACHE, Acute Physiology and Chronic Health Evaluation; HIV, human immunodeficiency.

### Fluid balance and process of care variables

**Figure 1:**
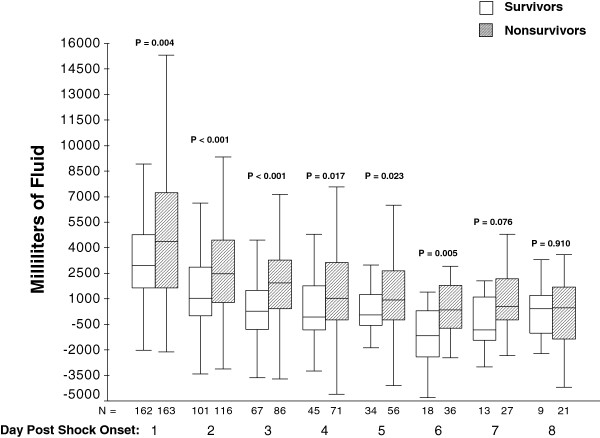
**Box plots depicting daily cumulative fluid balance for survivors (white boxes) and non-surviviors (hatched boxes).** The lines within the boxes represented the 50^th^ percentile, the lines at the bottom and top of the boxes represent the 25^th^ and 75^th^ percentiles, and the whisker lines represent the 5^th^ and 95^th^ percentiles.

Table 2 outlines the process of care variables. Non-survivors had a larger net fluid balance within the 24-hour window of septic shock onset (Table 2). Non-survivors also had a larger net fluid balance within the eight-day window of septic shock onset (median (interquartile range (IQR)): 7,742 ml (2,914 ml, 15,992 ml) vs. 3,286.5 ml (1,508.5 ml, 7,467 ml), *P* <0.001). There was no difference in AIFR or CVP and S_CV_O_2_ measurements or attainment of a CVP ≥8 mmHg and S_CV_O_2_ ≥70%. Figure 1 shows that significantly greater daily net fluid balance occurred in non-survivors for days 1 through 6 following septic shock onset.

**Table 2:** Process of care variables

**Variable**	**Survivors**	**Nonsurvivors**	***P-*** **value**
	**N = 162**	**N = 163**	
Fluid balance (ml), within 24 hours of septic shock onset	2,959	4,374	0.004
	(1,639.5,4,769.5)	(1,637,7,260)	
Fluid balance (ml/kg), within 24 hours of septic shock onset	37.5	53.3	0.022
	(20.8,62.2)	(19.8,91.7)	
Adequate initial fluid resuscitation, n(%):*	103 (63.6)	109 (66.9)	0.533
CVP measured, n(%):*	146 (90.1)	147 (90.2)	0.985
CVP ≥8 mm Hg, n(%):*	138 (94.5)	143 (97.3)	0.256
S_CV_O_2_ measured, n(%):*	69 (42.6)	60 (36.8)	0.287
S_CV_O_2_ ≥70%, n(%):*	62 (89.9)	50 (83.3)	0.275
PRBC administered, n(%):*	21 (13.0)	31 (19.0)	0.137
Vasopressor and inotrope usage, n(%):			
Norepinephrine	162 (100.0)	163 (100.0)	1.000
Dopamine	12 (7.4)	16 (9.8)	0.439
Vasopressin	9 (5.6)	34 (20.9)	<0.001
Epinephrine	9 (5.6)	24 (14.7)	0.006
Dobutamine	34 (21.0)	50 (30.7)	0.046
Requiring vasopressor support at Day 8 post septic shock onset, n(%):	9 (5.6)	21 (12.9)	0.002
Left ventricular dysfunction, n(%):			
Mild	22 (13.6)	10 (6.1)	0.024
Moderate	15 (9.3)	15 (9.2)	0.986
Severe	8 (4.9)	7 (4.3)	0.782
Left ventricle ejection fraction, n(%):	55 (49,60)	55 (55,70)	0.038
	(53.6 ± 12.3)	(56.5 ± 11.8)	
Right ventricular dysfunction, n(%):			
Mild	27 (16.7)	31 (19.0)	0.580
Moderate	6 (3.7)	13 (8.0)	0.101
Severe	1 (0.6)	1 (0.6)	1.000
Diastolic dysfunction	56 (34.6)	45 (27.6)	0.175
Appropriate initial antibiotic therapy, n(%):	136 (84.0)	125 (76.7)	0.364
Corticosteroids, n(%):	51 (31.5)	83 (50.9)	<0.001

Values are expressed as median with inter-quartile range. CVP, central venous pressure; PRBC, packed red blood cell; S_CV_O_2,_ central venous hemoglobin oxygen saturation.

*Within 24 hours of the onset of septic shock.

**Figure 2:**
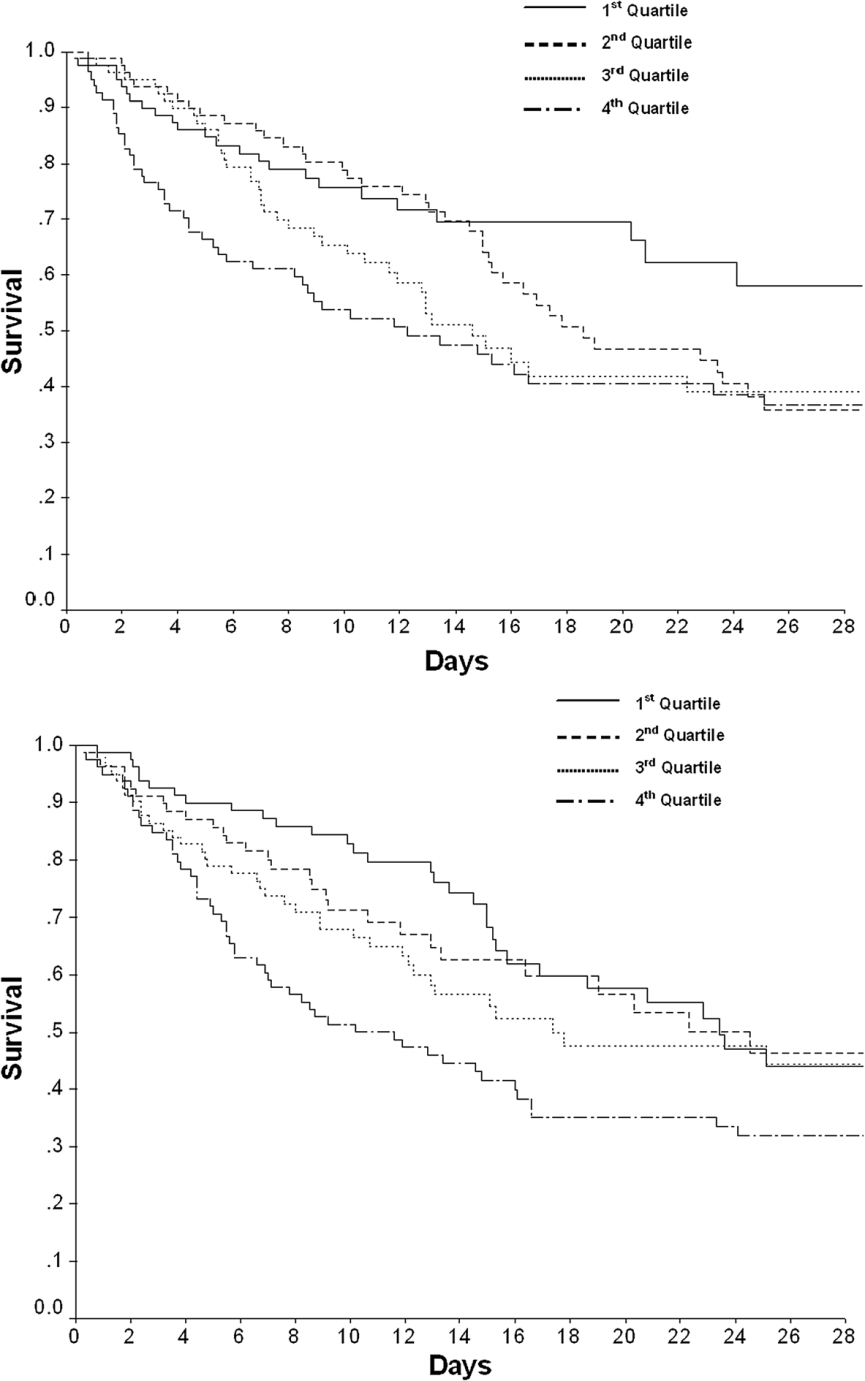
**Cox survival curves, adjusted for age, APACHE II scores, and the use of vasopressin are shown for fluid balance quartiles, 24 hours (Top) and at Day 8 (Bottom).** Quartile 4 has significantly decreased survival risk compared to quartiles 1 and 2 at 24 hours and 8 days, respectively.

Survival curves adjusted for age, APACHE II scores and vasopressin use are shown in Figure 2. Both at 24 hours and at Day 8, one’s fluid balance quartile predicted survival. At 24 hours, compared with quartiles 1 and 2, the risk of survival in Quartile 4 was significantly lower (*P* = 0.001 and *P* = 0.034, respectively, by log-rank test) (*P* = 0.162 for quartile 4 compared to Quartile 3) (Figure 2, Top). At eight days, compared with quartiles 1 and 2, the risk of survival in Quartile 4 was significantly lower (*P* <0.001 and *P* = 0.008, respectively, by log-rank test) (*P* = 0.60 for Quartile 4 compared to Quartile 3) (Figure 2, Bottom).

All patients received norepinephrine (Table 2). Non-survivors were statistically more likely to also receive vasopressin, epinephrine and dobutamine compared to survivors. The median (IQR) duration of vasopressor use was significantly longer among non-survivors compared to survivors (three days (one day, five days) versus two days (one day, four days); *P* = 0.006). Net fluid balance was significantly greater in patients receiving norepinephrine plus dobutamine, dopamine, epinephrine or vasopressin compared to norepinephrine alone (median (IQR): 4,194 ml (1,650.5 ml, 7,296 ml) vs. 3,147 ml (1,560.5 ml, 5,385.5 ml), *P* = 0.029). There was also a modest correlation between CVP measured within 24 hours of shock onset and net fluid balance at 24 hours (*P* = 0.033).

**Figure 3:**
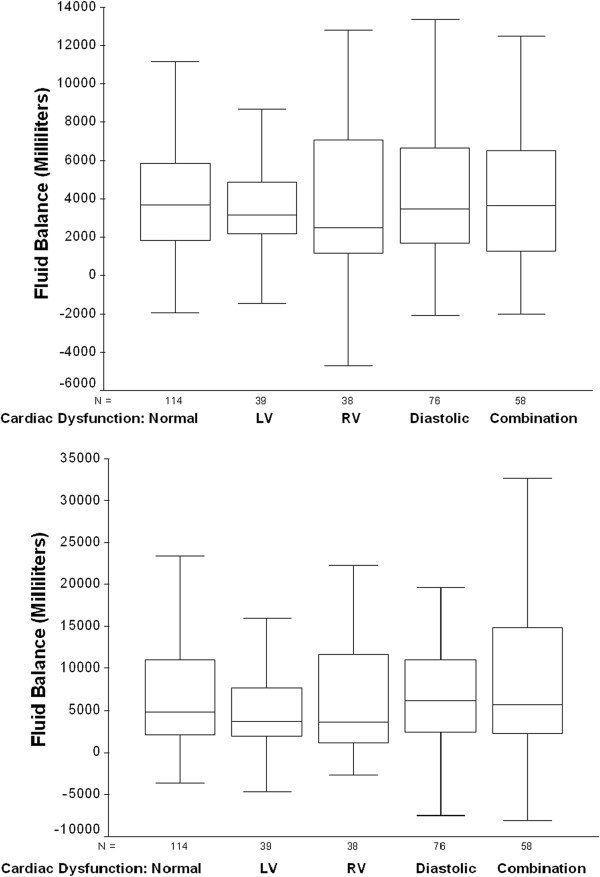
**Box plots depicting cumulative fluid balance at 24 hours (Top) and 8 days (Bottom) following shock onset for patients with and without cardiac dysfunction demonstrated by echocardiographic examinations.** LV, left ventricle; RV, right ventricle.

Cardiac function based on echocardiographic examinations is shown in Table 2. One hundred fourteen (35.1%) patients had normal examinations, 39 (12.0%) had LV dysfunction alone, 38 (11.7%) had RV dysfunction alone, 76 (23.4%) had diastolic dysfunction alone, and 58 (17.8%) had a combination of LV, RV and diastolic dysfunction. Mild LV dysfunction was statistically more common in survivors compared to non-survivors and LVEF was statistically higher among the non-survivors, although the median value was the same for both groups. Hospital mortality was 53.5% for patients with normal echocardiographic examinations and 35.9% for those with isolated LV dysfunction (*P* = 0.058 compared to normal), 60.5% for those with isolated RV dysfunction (*P* = 0.451 compared to normal), 48.7% for those with isolated diastolic dysfunction (*P* = 0.514 compared to normal), and 48.3% for those with a combination of LV, RV and diastolic dysfunction (*P* = 0.516 compared to normal). Cumulative fluid balance was similar at 24 hours and 8 days following the onset of septic shock for patients with and without cardiac dysfunction (Figure 3).

### Outcomes and multivariate analysis

ICU and hospital lengths of stay were significantly greater among non-survivors compared to survivors (ICU; 5.8 days (2.8 days, 11.6 days) vs. 4.4 days (2.0 days, 8.8 days), *P* = 0.016) (Hospital; 14.5 days (8.7 days, 27.8 days) vs. 7.3 days (3.4 days, 15.0 days), *P* <0.001). Multivariate analysis identified increasing APACHE II scores, age, LVEF and the greatest quartile of positive net fluid balance at eight days post-shock onset as independent risk factors for hospital mortality (Table 3). Similarly, the propensity score analysis found the greatest quartile of positive net fluid balance at eight days post-shock onset (Quartile 4) to be significantly associated with greater mortality (adjusted odds ratio (AOR) = 1.34; 95% CI = 1.19 to 1.50; *P* = 0.013).

**Table 3:** Multivariate analysis of independent risk factors for hospital mortality*

	**Adjusted odds ratio**	**95% Cl**	**P**
APACHE II score (one-point increments)	1.05	1.03 to 1.07	0.035
Age (one-year increments)	1.02	1.01 to 1.03	0.028
Left ventricle ejection fraction (one-point increments)	1.04	1.02 to 1.06	0.025
Greatest quartile of positive net fluid balance at eight days post-shock onset (quartile 4)	1.66	1.39 to 1.98	0.004

*Other covariates not in the table had a *P*-value >0.05 including use of dobutamine, vasopressin or epinephrine; presence of abnormal left ventricle function; presence of abnormal right ventricle function; diastolic dysfunction; 24-hour cumulative fluid balance quartiles; and inappropriate antibiotic therapy (Hosmer-Lemeshow goodness-of-fit test, *P* = 0.422). APACHE, Acute Physiology and Chronic Health Evaluation.

## Discussion

Our study demonstrated that daily and overall cumulative fluid balance predicts outcome in patients with septic shock. We also found a dose–response relationship between 24-hour and 8-day net fluid balance quartiles and hospital mortality. The greatest quartile of positive net fluid balance at eight days post-shock onset was also found to have the greatest adjusted odds ratio associated with hospital mortality in our multivariate analysis. LVEF was also a predictor of outcome.

Our results are consistent with those of Boyd *et al*. who showed that more positive fluid balance both early in resuscitation and cumulatively over four days was associated with an increased risk of mortality in septic shock [10]. These findings are consistent in showing that early and late net fluid balance assessed as quartiles of fluid balance predict hospital mortality. The daily differences in fluid balance from our study also correlated with outcome through Day 6, similar to our earlier study of septic shock complicated by ALI [9]. Boyd *et al*. also found that CVP predicted mortality at 12 hours following septic shock but not thereafter [10]. We did not find any independent predictive value in the CVP values obtained within the 24-hour window following septic shock onset, although a modest correlation between CVP and cumulative fluid balance in the first 24 hours was observed.

Other investigators have found associations between fluid balance and outcome in septic patients. Cordemans *et al*. observed that fluid balance and extravascular lung water index were predictors of mortality in critically ill patients requiring mechanical ventilation [11]. Maitland *et al*. studied children with severe febrile illness and impaired perfusion in resource-limited African countries who received either intravenous fluid boluses (20 to 40 mg/kg of body weight) or no fluid bolus [23]. Fluid bolus administration was associated with significantly increased 48-hour and 4-week mortality. A recent investigation evaluating critically ill cancer patients, many of whom had underlying infections, also found that positive fluid balance was independently associated with mortality [24].

The mechanisms by which positive fluid balance can adversely influence outcomes are not known. However, prolonging time on mechanical ventilation, due to positive fluid balance and increased lung water, could contribute to the development of nosocomial infections and other adverse outcomes [8,25,26]. The interruption of genetically-determined catecholamine-mediated host defense responses by the rapid increase in plasma volume might result in a reperfusion injury [27]. Additionally, hypervolemia or hyperosmolarity might exacerbate capillary leaks in patients with septic shock contributing to intracranial hypertension or pulmonary edema [28]. Positive fluid balance could also result in intra-abdominal hypertension contributing to the development of organ hypoperfusion and subsequent organ failure [5,29].

We also observed that more normal LV function and slightly greater LVEF were associated with a greater risk of mortality. These results are consistent with those of other investigators who showed that LV systolic dysfunction was associated with improved 28-day all-cause mortality in sepsis [12,30]. Many hypotheses have been proposed for myocardial depression in septic shock. However, most of them could not explain why survivors exhibited more marked myocardial depression [12,30]. Levy *et al.* documented myocardial hibernation to be present in sepsis by using magnetic resonance imaging, positron emission tomography and single-photon emission computed tomography imaging [31]. Myocardial hibernation is an adaptive response to maintain myocardial viability for prevention of cell-death pathway activation and preserves cardiac myocytes by down-regulation of oxygen consumption and energy requirements. Persistent vasoplegia is another potential explanation for our findings. The same level of LVEF may correspond to very different levels of intrinsic LV contractility [32]. For instance, normal values for LVEF may correspond to more severely impaired LV contractility in the presence of decreased vascular tone. This is supported by our observation that non-survivors required more vasopressors for a longer period of time, yet had slightly greater values for LVEF. The presence of a hyperkinetic state during sepsis associated with persistent and profound vasoplegia could represent the presence of uncontrolled infection and sustained inflammation [12].

Our study has several important limitations. First, it was performed in a large teaching hospital and may not be generalizable to other types of institutions. However, the results are consistent with those demonstrated by other investigations suggesting that these findings are more generalizable [9-11,23-26,33]. Second, the retrospective study design limits our ability to determine a causal relationship between fluid management and the outcomes we evaluated. Third, a formal protocol for fluid management of septic shock was present but limited to the initial administration of a fluid bolus of 20 ml/kg, with subsequent fluid therapy provided by goal-directed parameters [17,18]. The validity of this approach awaits the results of ongoing prospective trials. Fourth, we did not routinely utilize direct or indirect measures of stroke volume to guide our fluid therapy. Finally, despite achieving AIFR and appropriate antibiotic therapy in the majority of our patients, we cannot be certain that some other unmeasured clinical parameter or process of care variable may have contributed to our findings.

## Conclusions

The fluid management and cardiac function of patients with septic shock appear to be important potentially modifiable determinants of hospital mortality. These data support the need for prospective trials aimed at identifying the optimal strategies for hemodynamic management of septic shock to include fluid administration and cardiac support measures.

## Key messages

•Cumulative fluid balance and cardiac function predict outcome in patients with septic shock.

•Clinicians treating patients with septic shock should carefully assess the need for intravenous fluids both in the immediate resuscitation period and over the subsequent days of treatment.

•The use of more conservative fluid administration protocols in patients with severe sepsis and septic shock needs additional study to determine their relative efficacy compared to standard of care therapy.

## Abbreviations

AIFR: Adequate initial fluid resuscitation; ALI: Acute lung injury; AOR: Adjusted odds ratio; APACHE: Acute physiology and chronic health Evaluation; CI: Confidence interval; CVP: Central venous pressure; ICU: Intensive care unit; IQR: Interquartile range; LV: Left ventricle; LVEF: Left ventricular ejection fraction; RV: Right ventricle; SCVO2: Central venous oxygen saturation; SD: Standard deviation; TDI: Tissue Doppler imaging.

## Competing interests

Dr. Kollef’s effort was supported by the Barnes-Jewish Hospital Foundation. The authors have no conflicts of interest to report in relation to this manuscript.

## Authors’ contributions

MHK, CM, MM, NH, JAD and STM had full access to all of the data in the study and take responsibility for the integrity of the data and the accuracy of the data analysis. MHK and CM contributed to the study conception and design, statistical analysis, drafting of the manuscript, and have given approval to the final version. MM and NH contributed to the study conception and design, statistical analysis, and have given approval to the final version. JAD contributed to the study conception and database construction, and drafting of the manuscript and has given approval to the final version. STM contributed to the study conception and design, statistical analysis, and drafting of the manuscript and has given approval to the final version. All authors read and approved the final manuscript.
